# Impact of the expanded examination of fetal heart to the prenatal diagnosis of congenital heart diseases

**DOI:** 10.4274/tjod.galenos.2020.04127

**Published:** 2020-12-10

**Authors:** Pelin Koşger, Melih Velipaşaoğlu, Tuğçem Keskin, Hikmet Kıztanır, Birsen Uçar

**Affiliations:** 1 Eskişehir Osmangazi University Faculty of Medicine, Department of Pediatric Cardiology, Eskişehir, Turkey; 2 Eskişehir Osmangazi University Faculty of Medicine, Department of Obstetrics and Gynecology, Perinatology Unit, Eskişehir, Turkey; 3 Eskişehir State Hospital, Clinic of Pediatric Cardiology, Eskişehir, Turkey

**Keywords:** Congenital heart disease, fetal echocardiography, high risk pregnancy, low risk pregnancy, prenatal diagnosis

## Abstract

**Objective:**

In the present study, for which reasons fetal cardiac evaluation was requested from our pediatric cardiology clinic, the effects of routine fetal cardiac evaluation in obstetric ultrasonography (USG) on the detection of congenital heart disease (CHD) and the distribution of intrauterine diagnosis of CHD according to pregnancy risk profiles were retrospectively analyzed.

**Materials and Methods:**

Fetal echocardiography reports which containing the nineteen-month period were retrospectively examined. We performed a fetal echocardiography for all pregnant women who were referred to pediatric cardiology clinic after detail obstetric USG screening. The pregnancies were categorized into two groups based on the risk of CHD: Low-risk and high-risk groups. Detected congenital cardiac structural malformations were classified as complex, moderate, and mild according to perinatal mortality risk.

**Results:**

Of the 736 pregnancies, 22 were twin, and fetal cardiac evaluation was performed in 758 fetuses. There were 341 (46.3%) pregnancies in the high-risk group and 395 (53.6%) pregnancies in the low-risk group. The most common reason for fetal cardiac evaluation request was inability to adequately visualize the fetal heart (36.1%), while suspected fetal cardiac abnormality was the second most common cause (21.3%). Number of fetuses detected with cardiac abnormalities was 80 (23.5%) among high-risk pregnancies, and 20 (5%) among low-risk pregnancies. The most common type of malformation was simple cardiac abnormalities (6%) followed by complex lesions (4.1%). The most common cardiac abnormality was ventricular septal defect comprised of 18 cases (2.4%) while the most common complex cardiac abnormality was pulmonary atresia (1.2%). The rate of consistency was 40.1% between obstetricians and pediatric cardiologist in terms of the diagnosis of the congenital cardiac malformations.

**Conclusion:**

Routine evaluation of the fetal heart by means of obstetric USG, including four chambers, outflow tracts’ and three vessel views, would allow for diagnosing congenital cardiac malformations to a large extent during the intrauterine period.

**PRECIS:** The expanded examination of the fetal heart by means of obstetric USG increases the rate of prenatal detection of congenital heart defects.

## Introduction

Congenital heart disease (CHD) is the most common congenital abnormality, and it is six times more common than chromosomal abnormalities and four times more common than neural tube defect^(1)^. The incidence of CHD is 8-10 cases per 1000 live births (0.8%-1%) for full-term births, and this rate is approximately 10 times more (8.3%) for preterm births^(2)^. Approximately 17%-33% of CHDs include critical malformations, which must be intervened directly after birth and/or just before birth. Unfortunately, 50% of these malformations are diagnosed after the infant is discharged from the hospital, and the mortality risk increases because of the delay. The identification of severe cardiac abnormalities during the intrauterine period enables the use of approaches that lead to significant decreases in perinatal morbidity and mortality, such as performing the delivery at a center where cardiac surgery can be performed, providing the required medical support in the newborn intensive care unit until transfer to the relevant center, and/or if needed, promptly performing transcatheter palliative interventions. Therefore, fetal cardiac evaluation has become an important part of obstetric ultrasonography (USG), and fetal echocardiography evaluation requests have increased due to increased incidences of suspected CHD. Although the risks of CHD are defined and fetal cardiac evaluation is recommended for all high-risk pregnancies, 90% of CHDs are observed in low-risk pregnancies^(3,4)^.

The current approach in many clinics in Turkey involves performing fetal cardiac evaluations within defined indications owing to long examination times and inadequate number of pediatric cardiologists and cardiovascular surgery experts. Our hospital is a tertiary center and includes the only perinatology and pediatric cardiology clinic that provides services to a large region, which consists of our city and neighboring cities. In the present study, for which reasons fetal cardiac evaluation was requested from our pediatric cardiology clinic, the effects of routine fetal cardiac evaluation in obstetric USG on the detection of CHDs and the distribution of intrauterine diagnosis of CHDs according to pregnancy risk profiles were retrospectively analyzed.

## Materials and Methods

In this retrospective study, the total number of anomaly scans in the perinatology unit during the study period (May 2017 - January 2019) were 8372 and the number of referred pregnancies for fetal echocardiography evaluations were 736. All patients in the study underwent routine fetal anatomic scanning according to the International Society of Ultrasound in Obstetrics and Gynecology guidelines between the 18^th^ and 22^nd^ weeks of pregnancy in the Department of Perinatology at our university. In this scan, four chambers of the heart, three vessel and trachea, left and right ventricular outflows, and the heart were examined using color Doppler USG^(5)^. Subsequently, the patients were referred to the Department of Pediatric Cardiology unit form perinatology division of our university hospital either suspected cardiac abnormalities, accompanying diseases that increase risk of fetal cardiac malformations, parental congenital heart malformation or suboptimal evaluation during routine anomaly scan. To identify all reasons for fetal echocardiography requests, detailed obstetric USG reports of each case were retrieved from the system and examined, and the reasons for fetal echocardiography requests were determined as accurately as possible by comparing these with our records. Pre-gestational and gestational medical data of pregnant women were retrieved. Comorbid medical problems that may be considered as risky for CHD, medication use, and characteristics of previous pregnancies were recorded. The pregnancies were categorized into two groups based on the risk of CHD: low-risk and high-risk groups. Fetal cardiac evaluation was performed by the same single operator  (P.K) in the Department of Pediatric Cardiology using a C5-1 convex probe with an Affinity 70 (Philips, WA, USA) device. In all fetal echocardiography examinations, cardiac situs, size and position, systemic and pulmonary venous connections, atrial and ventricular chambers, atrioventricular and semilunar valves, ventriculoatrial shunts, great vessels outflow tracts, ductal and aortic arches, and rhythm were evaluated. If all views could not be clearly evaluated or if a suspicious finding is detected in the first evaluation, repeated evaluations may be required. Echocardiography images and reports of each patient were recorded. Fetal echocardiographic evaluation was scheduled according to the gestational week of the pregnancy, the referral diagnosis of the fetus, and the availability of our out-patient clinic. Each case was informed about applying for postnatal cardiac evaluation even if no pathology was detected in the fetal evaluation. For the purpose of performing as many evaluations and as early as possible, postnatal echocardiography evaluations are performed without appointment in our clinic. The postnatal data of 261 infants were retrieved of 714 single and 22 sets of twins, and 758 infants in total from 736 pregnancies were examined. Prenatal and postnatal echocardiography data of 261 cases, for which postnatal data could be retrieved, were compared.

Detected congenital cardiac structural malformations were classified as complex, moderate, and mild according to perinatal mortality risk (Table 1)^(6,7)^. Malformations that are not included in this classification, and malformations with low mortality risk were evaluated in the others category. This study was approved by the Eskişehir Osmangazi University Institutional Ethics Committee.

### Statistical Analysis

Statistical analysis was performed using Statistical Package for Social Sciences, version 15 (SPSS, Chicago, USA). The pregnancies were categorized into two groups based on the risk of CHD. The fetal echocardiography request reasons and the congenital cardiac malformation rates were classified according to pregnancy risk profile. The prevalence of CHDs in low- and high- risk pregnancies was compared using the chi-square test. Statistical significance was inferred at p<0.05.

## Results

The mean age of the mothers was 29.8±5.6 years (range, 17-47 years), and the mean gestational age at which the fetal echocardiography examination was performed was 26.4±4.4 weeks. Of the 736 pregnancies, 22 were twin pregnancies, and fetal cardiac evaluation was performed in 758 fetuses. Twelve patients underwent examination for a second time. In total, 37.9% of the examinations were performed before the 24^th^ week of pregnancy, while 62.1% were performed after the 24^th^ week of pregnancy. Number of fetuses with congenital structural cardiac malformation was 101 (13.7%), and the number of fetuses with arrhythmia was 15 (2%).

The reasons for fetal echocardiography requests were classified according to CHD risk profiles considering the American Heart Association recommendations (Table 2)^(8)^. The prevalence of CHD according to risk factors was determined. There were 341 (46.3%) pregnancies in the high-risk group and 395 (53.6%) pregnancies in the low-risk group. The most common cause for fetal cardiac evaluation request was inability to adequately visualize the fetal heart (36.1%), while suspected fetal cardiac abnormality was the second most common cause (21.3%). Fetal cardiac malformation was detected as the most frequent among pregnant women referred to the pediatric cardiology due to suboptimal evaluation during routine anomaly scan (37.5%).

The distribution of CHDs, which were diagnosed with fetal echocardiography and classified according to mortality risk according to pregnancy risk groups was presented in Table 3. Number of fetuses detected with cardiac abnormalities was 80 (23.5%) among high-risk pregnancies, and 20 (5%) among low-risk pregnancies. The prevalence of cardiac abnormalities in each category was higher in high-risk pregnancies (Table 4). The most common type of malformation was simple cardiac abnormalities (6%) followed by complex lesions (4.1%). The most common cardiac abnormality was ventricular septal defect (VSD) comprised of 18 cases (2.4%) while the most common complex cardiac abnormality was pulmonary atresia (PA) (1.2%). The moderate cardiac abnormality was TOF including nine cases (1.2%). Out of 20 fetuses (2.7%) evaluated with suspected fetal cardiac arrhythmia, two had complete atrioventricular block, two fetuses had blocked premature atrial contractions, and two fetuses had supraventricular tachycardia. The other nine fetuses had premature atrial and ventricular contractions, which recovered during the late weeks of pregnancies.

The number of fetuses for which postnatal cardiac evaluation records could be retrieved via echocardiography examinations performed after birth, autopsy reports of terminated fetuses, and late period presentations to our clinic for follow-up purposes was 261 (35.4%). Of the 261 infants, 43 (16.4%) had CHD. As the prenatal and postnatal results were compared, there were 24 (9.1%) discordant diagnoses: One major and 23 minor. In one fetus for which fetal echocardiography was requested because of the presence of a sibling with heart disease and which did not have fetal cardiac abnormalities, total anomalous pulmonary venous return was detected in the first postnatal week. Twenty-three minor discordant diagnoses [15 atrial septal defect (ASD)], 4 VSD, 1 bicuspid aortic valve, 3 pulmonary stenosis] belonged to the class of simple heart diseases.

## Discussion

Diagnosis of CHD during the intrauterine period provides significant benefits as performing of the birth under appropriate conditions, mentally prepared family, defining potential genetic abnormalities, and termination of pregnancy in the presence of complex malformations. Moreover, it was reported that diagnosis of some specific CHDs during the prenatal period increases the survival rate as well^(9)^. However, due to long duration of fetal echocardiography and requirement of an experienced pediatric cardiologists, it is not routinely performed in all pregnant women. Thus, basic fetal cardiac evaluation has become a part of the USG in routine obstetric monitoring. CHDs can be detected during the intrauterine period at a rate of 4.5%-8.1% with the evaluation of the fetal heart in four chamber view and at 43.8%-85.5% with the additional examination of the right and left ventricular outflow tracts^(9)^. Therefore, the prevalence of intrauterine diagnosis of CHD varies according to the protocol performed by the centers for fetal cardiac evaluation^(10,11)^. In the present study consisting of an eighteen-month period, fetal congenital cardiac malformation prevalence was 13.7%. Fetal congenital cardiac malformation prevalence was reported to be 5.6% from another tertiary center in Turkey^(12)^. At our center, evaluation of the fetal heart in four chamber view is a routine part of the obstetric USG. Moreover, detailed fetal cardiac evaluation including right and left ventricular outflow tracts and three vessels and trachea view is routinely performed in each pregnant woman by skilled perinatologists between the 18^th^ and 22^nd^ weeks of pregnancy. Furthermore, since the fetal echocardiography was performed in selected pregnancies who were identified as risky in antenatal screening in our tertiary reference center, the reported prevalence may be higher than expected.

In the present study, while suspected fetal cardiac abnormality was the second most common (21.3%) reason for fetal echocardiography request, inadequate evaluation of the fetal heart was the most common reason (36.1%). Prevalence of family history of CHD and maternal diabetes mellitus (DM), which were reported to be the top two most common reasons for fetal echocardiography requests in the previous studies, were 3.4% and 7.8%, respectively in the present study^(10,13)^. In the present study, similar to Cha et al.^(14)^, detection of an abnormal cardiac finding during obstetric follow-up was more common than family history of CHD and maternal DM among the reasons for fetal echocardiography requests. Compatibility between the findings of the pediatric cardiologist and the obstetrician in the cases referred to fetal echocardiography with suspected CHD by the obstetricians varies according to the experience of the centers. In the current study, the rate of consistency was 40.1% between obstetricians and pediatric cardiologist in terms of the diagnosis of the congenital cardiac malformations. Simpson^(15)^ reported that cardiac malformation was detected during fetal echocardiography in 45 of 275 (16%) pregnant women referred with suspected CHD, while Meyer-Wittkopf et al.^(16) ^demonstrated that cardiac abnormality was detected in 209 out of 268 (77.9%) pregnant women referred to fetal echocardiography with suspected fetal cardiac abnormality, and the diagnosis was fully compatible in 62% of these patients. Obstetricians’ increasing experience in evaluating fetal heart enables us to diagnose a higher rate of CHD during the intrauterine period.

When pregnant women that underwent fetal echocardiography were classified according to risk levels in terms of fetal cardiac malformation; 46.3% were in the high-risk group, and 53.6% were in the low-risk group. In fetal echocardiography, CHD was detected most commonly in the high-risk group with a rate of 23.5%. In the low-risk group, determination of congenital heart abnormality rate was 5%, and it was significantly higher among high-risk pregnancies. This was associated with the detection of cardiac malformation in 40.1% of pregnancies referred to fetal echocardiography due to suspected fetal cardiac malformation in the obstetric USG. In the study of Nayak et al.^(9)^, in which fetal echocardiography was performed in all pregnant women over a period of 4 years, they highlighted the importance of fetal echocardiography. They concluded that the fetal echocardiography should be included as a part of routine antenatal screening irrespective of risk factors for CHD. According to the their results, the prevalence of prenatal CHD was similar between high- and low-risk pregnancies, but the majority of pregnancies with cardiac malformation was in the low-risk group^(9)^, which is contrary to our finding. Operator misevaluation, failure to notice cardiac malformation, and inadequacy of and failure to interpret fetal cardiac image views were given as the reasons for failure to detect complex-structured fetal cardiac malformations in obstetric USG. The missing diagnoses in these pregnancies, which were referred with low-risk, were made with fetal echocardiography^(9)^. Evaluation of fetal heart in obstetric USG requires experience and knowledge, and obstetric fetal cardiac evaluation which is not optimally performed owing to various reasons can cause complex cardiac malformations to be missed. Therefore, we find it appropriate to refer these pregnant women in whom fetal heart could not be adequately evaluated by obstetric USG with reasons similar to those stated by Nayak et al.^(9)^ to fetal echocardiography in our center as well. According to our results, inadequate evaluation of fetal heart in obstetric USG is the most common (31%) reason for requesting fetal echocardiography. However, fetal cardiac malformation was detected only in 4.1%. This was associated to a large extent with high-risk pregnancies in which obstetric fetal cardiac evaluation was conducted by an experienced perinatologist.

It is known that the prevalence of CHD differs during the intrauterine and postnatal periods. Isolated VSD is the most common CHD during the postnatal period, which is also reported to be the most common CHD diagnosed during the intrauterine period as well ^(12,17)^. In the present study, consistent with the literature, the most common cardiac malformation in fetal echocardiography is VSD. In the previous studies, complex cardiac malformations were reported to be the most common group of CHD^(9,12,18)^. However, we found complex cardiac malformations as the second most common following minor lesions. It was considered that this variation may be related to some differences in the classification of cardiac lesions between the studies. Moreover, in the present study, consideration of the higher but insignificant increases in flow velocities at semilunar valves may have played a role in the increased number of minor lesions. In previous studies, atrioventricular septal defect (AVSD) and hypoplastic left heart syndrome (HLHS) were reported to be complex cardiac lesions that are detected at a similar or higher prevalence^(12,18,19)^. In the present study, the most common complex CHD was PA, followed by HLHS.

Since the pregnancies diagnosed with complex type congenital cardiac malformations were referred to the surgical center during the prenatal or postnatal period, and the others diagnosed as moderate type lesions gave birth in our hospital, postnatal results of all of these pregnancies were retrieved. Moreover, most of the 261 (35.4%) cases, whose postnatal echocardiography results could be retrieved, were diagnosed with fetal cardiac abnormality by an obstetrician or pediatric cardiologist. The incompatibility between prenatal and postnatal echocardiography in these 261 cases were mostly due to simple lesions. It was found that the diagnoses did not change in complex lesions which were detected by a pediatric cardiologist, but one case with a normal fetal echocardiography was diagnosed with total anomalous pulmonary venous return abnormality during the postnatal period. Pulmonary venous return abnormalities, small or moderate sized ventricular or ASD, and minor valve lesions cannot always be defined with fetal echocardiography, and are frequently diagnosed after birth^(8)^. Meyer-Wittkopf et al.^(16)^ also reported that a total anomalous pulmonary venous return abnormality diagnosed in the postnatal period could not be detected in the fetal echocardiography.

### Study Limitations

Although each evaluated pregnant woman was informed about the importance of postnatal echocardiography, a significant amount of postnatal echocardiographic evaluation, in which a statistical analysis for sensitivity and specificity could not be performed in order to make a confirmation or comparison.

## Conclusion

In many centers, regardless of CHD risk, it is still not possible to perform fetal echocardiography by a pediatric cardiologist to all pregnant women. Therefore, routine evaluation of the fetal heart by means of obstetric USG, including four chambers, outflow tracts’ and three vessel views, would allow for diagnosing congenital cardiac malformations to a large extent during the intrauterine period.

## Figures and Tables

**Table 1:**
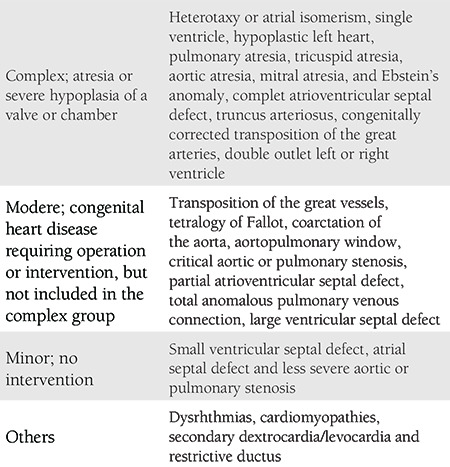
Classification of prenatally diagnosed congenital heart defects

**Table 2:**
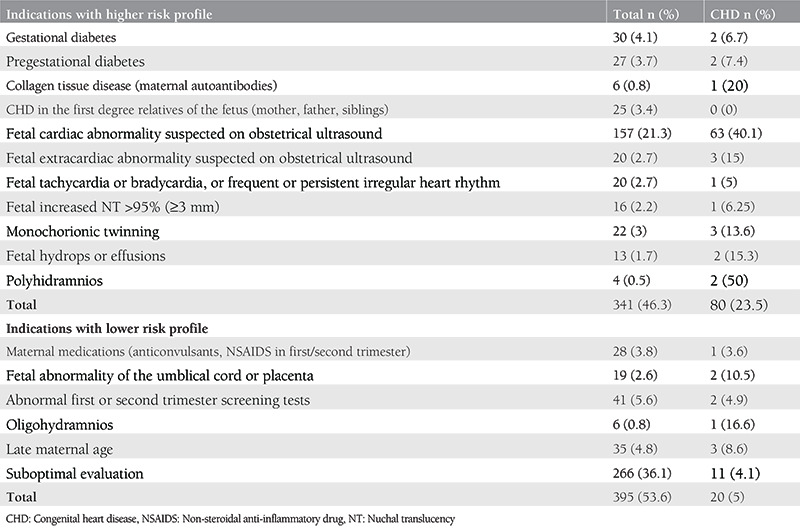
The distribution of the fetal echocardiography request reasons and the congenital cardiac malformation rates according to pregnancy risk profile*

**Table 3:**
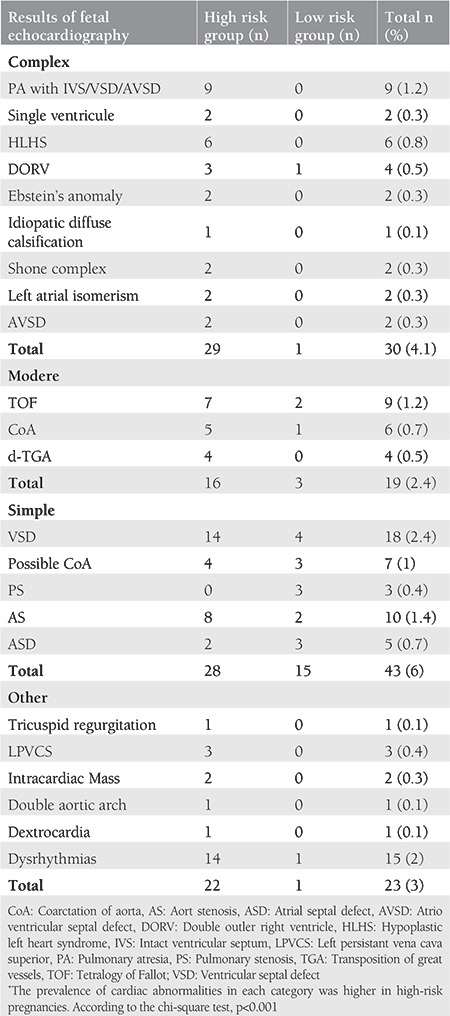
Intrauterine detected congenital heart diseases and their distribution according to pregnancy risk groups*

**Table 4:**
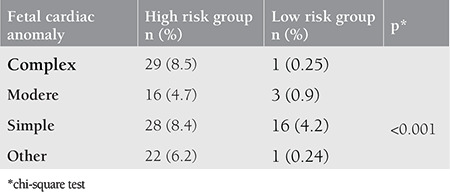
Comparison of pregnancy risk groups in terms of fetal echocardiography results
